# Disparities in health care outcomes between immigrants and the majority population in Germany: A trend analysis, 2006–2014

**DOI:** 10.1371/journal.pone.0191732

**Published:** 2018-01-23

**Authors:** Patrick Brzoska

**Affiliations:** Chemnitz Technical University, Faculty of Behavioral and Social Sciences, Chemnitz, Germany; National Institute of Health, ITALY

## Abstract

**Background:**

Immigrants often encounter barriers in the health system that may affect their health care outcomes. In order to better cater to the needs of immigrants, many health care institutions have increased their efforts in recent years to provide services which are more sensitive to the needs of an increasingly diverse population. Little is known about whether these efforts are successful. This study examines difference in outcomes of tertiary prevention between immigrants and the autochthonous population in Germany over the period of 2006–2014.

**Methods:**

The analysis is based on a 10% random sample of routine data on completed tertiary preventive treatments in Germany during 2006–2014. Four different indicators of treatment effectiveness were compared between patients with a nationality from Germany, Portugal/Spain/Italy/Greece, Turkey and Former Yugoslavia using logistic regression adjusted for demographic/socioeconomic factors. Interaction terms for year were modeled to examine group differences over time.

**Results:**

Depending on the outcome, Turkish and Former Yugoslavian nationals had an 23%-69% higher chance of a poor treatment effectiveness than Germans (OR = 1.23 [95%-CI = 1.15,1.32] and OR = 1.69 [95%-CI = 1.55,1.83], respectively). Fewer differences were observed between nationals from Portugal/Spain/Italy/Greece and Germans. Disparities did not significantly differ between the years in which services were utilized.

**Conclusion:**

Measures implemented by health care institutions did not reduce existing health care disparities between immigrants and the majority population in Germany. One potential reason is that existing approaches are unsystematic and often not properly evaluated. More targeted strategies and a thorough evaluation is needed in order to improve health care for immigrants sustainably.

## Introduction

In many European countries large proportions of the populations are immigrants. In Germany, more than 20% of the population is considered to be of immigrant origin. About half of them are non-German nationals, equaling about 7.6 million individuals. Turkish nationals, nationals from a former Yugoslavian country and from the South European countries Portugal, Spain, Italy and Greece make up the largest share of immigrants in Germany [1]. Immigrants often differ from the majority populations of the countries they reside in terms of health status, health behavior and health care outcomes. Particularly older immigrants tend to have a worse health status than the autochthonous population of the same age [2–4]. Aside from poor working conditions and a lower socioeconomic status, barriers immigrants encounter in the health system contribute to this differential by affecting the access to health care. This results in a lower utilization of preventive services such as screening [5–7], vaccination [8–10] and rehabilitative care [11–14]. These barriers may also contribute to disparities in health care outcomes between both population groups [11;15;16]. For example, a study from Germany found out that while 15.5% of all Germans who underwent rehabilitation in 2006 were reported to have a poor occupational performance after treatment, the respective proportions were considerably larger for non-German nationals, being 23.0% for Turkish nationals, 25.1% for Former Yugoslavian nationals and 19.6% for nationals from Portugal, Spain, Italy or Greece. This difference was independent of demographic and socioeconomic covariates [11]. Similar disparities were observed for other outcomes such as the self-rated treatment effectiveness and the risk for disability retirement after rehabilitation [15;17].

Different recommendations have been published on how health care of immigrants can be improved through the implementation of migrant- or diversity-sensitive measures, which aim to remove existing barriers and to facilitate a more patient-oriented health care [18–21]. In order to better cater to the needs of immigrants, also in Germany many health care institutions have increased their efforts in recent years to provide services which are more sensitive to the needs of an increasingly diverse population. These include, for instance, foreign language information material, cross-cultural trainings for health professionals as well as the employment of health navigators and interpreters [21–25]. Little is known about whether these efforts are successful and whether they translate into reducing health care disparities between immigrants and the majority population. Using rehabilitative care data from a large social security organization as an example, the present study examines whether difference in outcomes of health care between immigrants and the autochthonous population in Germany decreased over the period of 2006–2014. In addition, the study examines whether disparities are moderated by sex and age. The study findings can be informative for practice and research and may contribute to devising appropriate strategies aiming to reduce existing disparities in health care.

## Materials and methods

### Data

The study uses a 10% random sample of 2006–2014 annual cross-sectional routine data from the German Statutory Pension Insurance Scheme (Scientific Use File SUFRSDQJ0XX; no data for the year 2009 was available). It is based on insurance data according to §4 and §13 of the General Administrative Rule on Statistics in the Pension Insurance Scheme (*Allgemeinen Verwaltungsvorschrift über die Statistik in der Rentenversicherung [RSVwV]*) providing information on a sample of all subjects who completed a rehabilitative treatment covered by the German Statutory Pension Insurance Scheme (10% is the standard sample size provided by the German Statutory Pension Insurance Scheme for research purposes). The German Statutory Pension Insurance Scheme provides mandatory insurance for all employees who are subject to social insurance contributions. In general, this includes all wage earners and salaried employees. In terms of rehabilitation, the German Statutory Pension Insurance Scheme is responsible for all treatments conducted for patients in working age and for patients with cancer. This comprises about two-thirds of all rehabilitative treatments provided in Germany [26], which are usually conducted as in-patient programs in specialized hospitals [27]. Except for cancer cases, individuals not participating in the labor market such as pensioners are usually not provided with rehabilitative services from the German Statutory Pension Insurance Scheme and are therefore not included in the data used for the present study. No other exclusion criteria were set.

The present study uses administrative data which fulfils all necessary requirements and guidelines of the Federal data protection act of the Federal Republic of Germany and follows the requirements as defined by the German Social Code VI, IX and X. As the data is fully anonymous and did not involve any experiments, no further ethical approval was necessary according to national standards for secondary data analyses in Germany [28]. The data that support the findings of this study are available free of charge from the German Statutory Pension Insurance Scheme for researchers who meet the criteria for access to confidential data. Further information (only in German language) are available from http://forschung.deutsche-rentenversicherung.de/FdzPortalWeb/.

### Measures

Four different indicators of treatment effectiveness over eight time points (2006–2008 and 2010–2014) were examined. All outcomes were based on a medical evaluation at the time of completion of treatment [29]. The first outcome of interest was the *improvement in the health condition* diagnosed upon referral to rehabilitation (improved, not improved). The second outcome was the *presence of mental and physical impairment* after the treatment (no, yes). As an outcome of occupational performance after treatment the *number of hours per day the patient is able to follow an occupation* was considered. It is measured by means of the three categories ‘full-time’ (≥6 hours/day; full performance), ‘part-time’ (3 to <6 hours/day; medium performance) and ‘less than part-time’ (<3 hours/day; low performance). The present study distinguishes between patients who completed rehabilitation with a low and a medium/full performance following the procedure of previous research in the field [11]. As a second outcome of occupational performance the patient’s *work capacity* (capable of performing heavy/medium work, only capable of performing light work) was considered.

The four outcomes were compared between five population groups defined by citizenship: German nationals, Turkish nationals, nationals from a former Yugoslavian country, nationals from the South-Western European countries Portugal/Spain/Italy/Greece and nationals from other countries. Different confounding variables were taken into account which may be associated with the effectiveness of treatment and which may also differ between the population groups. The confounding variables were *age* (in years), *sex*, *marital status* (single/divorced/widowed, married, unknown), *occupational position* (skilled labor, semi-skilled/unskilled labor, trainee/unemployed), *employment status* (full-time, part-time, unemployed, not applicable) and the *type of occupation* (manual, services, technical/professional, administrative, other). Information on *type of diagnosis* upon referral to rehabilitation (diseases of the skeletal system, diseases of the circulatory system, neoplasms, mental conditions, other) and the *time absent from work due to illness in the last 12 months before rehabilitation* (0 months, <3 months, 3 to <6 months, ≥6 months, not employed) were considered as proxy variables for health status. The analysis was also adjusted for the *region of residence* and for the *region of treatment* (seventeen categories representing the sixteen German federal states and the category “unknown/living abroad”).

### Statistical analysis

Following a sample description by means of χ^2^- and Mann-Whitney-U-tests were appropriate, a multivariable logistic regression was conducted in order to adjust the comparison of the four outcomes between the population groups for confounding variables. Results are reported using adjusted odds ratios (aOR) and their 99% confidence intervals (99%-CI). Given that multiple outcomes are studied, a conservative significance level of α = 1% was used. In order to examine whether differences between the population groups vary with sex and age, sex- and age-interaction effects were included into the model. Similarly, interaction terms for year of completion of rehabilitation were modelled to examine whether differences between the population groups in terms of effectiveness of treatment change over time. Considering that unobserved heterogeneity may bias the evaluation of interaction terms based on odds ratios [30], average marginal effects are reported for the illustration of interaction effects. They represent differences in the predicted probability for the occurrence of the outcome. Analyses were conducted using Stata 13 [31].

## Results

Information on 617,683 subjects was available who were evaluated on their health status and occupational performance after completing their rehabilitative treatment in the years 2006 through 2014. Of these, 1.6% (n = 9,925) were nationals from Turkey, 1.1% (n = 6,943) had a nationality from a former Yugoslavian country, 1.1% (n = 6,851) held a nationality from the South European countries Portugal, Spain, Italy or Greece and 2.2% (n = 13,747) were nationals from other countries. In total, 6.1% (n = 37,466) of all subjects had a non-German nationality.

The five population groups differed in their demographic and socioeconomic characteristics (Table 1). Overall, the socioeconomic status of non-German nationals was lower than that of Germans as is reflected in a higher proportion of non-German nationals working in manufacturing positions and performing semi-skilled/unskilled labor. Non-German nationals had a slightly higher age (except for the group of ‘other’ non-Germans) and a lower male-female ratio. Also, the distribution of diseases that were diagnosed upon referral to rehabilitation differed between the population groups.

**Table 1:** Sample description by population group. (10% random sample of all individuals who completed medical rehabilitation in the years 2006–2008 and 2010–2014 granted by the German Statutory Pension Insurance Scheme; cases with available data on the four outcomes studied, n = 617,683).

	Germany (n = 580,217)		Portugal/Spain/Italy/Greece (n = 6,851)		Former Yugoslavia (n = 6,943)		Turkey (n = 9,925)		Other (n = 13,747)		p-value
**Sex** (n,%)											<0.001
Male	297,095	51.2	4,402	64.3	3,744	53.9	6,194	62.4	7,696	56.0	
Female	283,122	48.8	2,449	35.7	3,199	46.1	3,731	37.6	6,051	44.0	
**Age** (mean, sd)	49.4	9.5	50.0	9.1	50.6	9.9	45.9	9.3	48.9	9.7	
**Marital status** (n,%)											<0.001
Not married	201,620	34.7	1,732	25.3	1,556	22.4	1,934	19.5	4,178	30.4	
Married	364,751	62.9	5,067	74	5,330	76.8	7,924	79.8	9,333	67.9	
Other	13,846	2.4	52	0.8	57	0.8	67	0.7	236	1.7	
**Occupational position** (n,%)											<0.001
Skilled labor	431,553	74.4	3,444	50.3	3,358	48.4	4,023	40.5	8,124	59.1	
Semi-skilled/unskilled labor	94,978	16.4	2,979	43.5	3,072	44.2	5,003	50.4	4,344	31.6	
Trainee/unemployed	53,686	9.3	428	6.2	513	7.4	899	9.1	1,279	9.3	
**Employment status** (n,%)											<0.001
Fulltime	372,005	64.1	4,966	72.5	4,861	70.0	6,511	65.6	8,632	62.8	
Part-time	93,012	16.0	664	9.7	780	11.2	829	8.4	1,790	13.0	
Unemployed	53,690	9.3	718	10.5	705	10.2	1,559	15.7	1,913	13.9	
Other	61,510	10.6	503	7.3	597	8.6	1,026	10.3	1,412	10.3	
**Occupation** (n,%)											<0.001
Manual	156,428	27.0	3,328	48.6	3,186	45.9	4,814	48.5	4,638	33.7	
Services	130,617	22.5	2,129	31.1	2,381	34.3	3,133	31.6	4,193	30.5	
Technical/professional	89,852	15.5	286	4.2	312	4.5	339	3.4	1,401	10.2	
Administrative	99,484	17.1	453	6.6	375	5.4	594	6.0	1,167	8.5	
Other	103,836	17.9	655	9.6	689	9.9	1,045	10.5	2,348	17.1	
**Diagnosis** (n,%)											<0.001
Skeletal system	88,810	15.3	881	12.9	847	12.2	1,422	14.3	2,090	15.2	
Circulatory system	284,140	49.0	3,141	45.8	3,002	43.2	3,496	35.2	6,423	46.7	
Neoplasms	82,594	14.2	1,153	16.8	1,253	18	1,767	17.8	2,078	15.1	
Mental diseases	99,667	17.2	1,371	20	1,441	20.8	2,548	25.7	2,473	18	
Other	25,006	4.3	305	4.5	400	5.8	692	7.0	683	5.0	
**Time absent from work in the last 12 months** (n,%)											<0.001
None	284,409	49	3,482	50.8	3,814	54.9	4,486	45.2	6,786	49.4	
<3 months	55,106	9.5	808	11.8	697	10	1,108	11.2	1,629	11.8	
3–6 months	46,494	8	489	7.1	481	6.9	428	4.3	1,162	8.5	
6+ months	87,663	15.1	923	13.5	1,076	15.5	2,417	24.4	1,673	12.2	
Not employed	106,545	18.4	1,149	16.8	875	12.6	1,486	15	2,497	18.2	
**Improvement in health condition after rehabilitation** (n,%)											<0.001
Yes	490,551	84.5	5,581	81.5	5,400	77.8	7,533	75.9	11,340	82.5	
No	89,666	15.5	1,270	18.5	1,543	22.2	2,392	24.1	2,407	17.5	
**Presence of mental and physical impairment after rehabilitation** (n,%)											<0.001
No	537,546	92.6	6,249	91.2	6,114	88.1	8,475	85.4	12,742	92.7	
Yes	42,671	7.4	602	8.8	829	11.9	1,450	14.6	1,005	7.3	
**Limitations in occupational performance in terms of working hours per day (less then part-time) after rehabilitation** (n,%)											<0.001
No	493,294	85.0	5,562	81.2	5,256	75.7	7,624	76.8	11,386	82.8	
Yes	86,923	15.0	1,289	18.8	1,687	24.3	2,301	23.2	2,361	17.2	
**Limitations in occupational performance in terms of work capacity (only capable of performing light work) after rehabilitation** (n,%)											<0.001
No	482,611	83.2	5,658	82.6	5,479	78.9	8,089	81.5	11,513	83.7	
Yes	97,606	16.8	1,193	17.4	1,464	21.1	1,836	18.5	2,234	16.3	

*Note*. sd: Standard deviation

Among 15.5% of all German patients the health status did not improve through rehabilitation. This proportion was higher for the non-German population groups, being 18.5% for nationals from Portugal/Spain/Italy/Greece, 24.1% for Turkish nationals, 22.2% for nationals from a former Yugoslavian country and 17.5% for ‘other’ nationals. A poorer health status after rehabilitation was also reflected in a higher percentage of individuals among non-Germans who had limitations with respect to their mental and physical condition despite the treatment. Non-German nationals also completed rehabilitation with a poorer occupational performance. While 15.0% of Germans were only able to work less than part-time after rehabilitation, the percentages were 17.2% for ‘other’ nationals, 18.8% for nationals from Portugal/Spain/Italy/Greece, 23.2% for Turkish nationals and 24.3% for nationals from a former Yugoslavian country. A poorer occupational performance became also evident in limitations in terms of work capacity. Differences between the population groups with respect to the four outcomes only slightly varied between the years (Table 2).

**Table 2:** Indicators of poor treatment effectiveness by population group and years in which services were utilized. (10% random sample of all individuals who completed medical rehabilitation in the years 2006–2008 and 2010–2014 granted by the German Statutory Pension Insurance Scheme; cases with available data on the four outcomes studied, n = 617,683).

Outcome/Year	Germany (n = 580,217)	Portugal/Spain/Italy/Greece (n = 6,851)	Former Yugoslavia (n = 6,943)	Turkey (n = 9,925)	Other (n = 13,747
**No improvement in health condition after rehabilitation (%)**					
2006	15.5	17.6	22.9	24.6	17.1
2007	15.6	19.1	23.9	24.4	17.5
2008	15.6	16.8	19.6	23.9	16.9
2010	15.9	18.5	22.3	25.5	18.1
2011	15.8	16.0	23.8	23.7	17.9
2012	15.9	20.0	21.5	24.3	18.2
2013	15.9	20.8	24.5	25.8	17.9
2014	16.3	21.4	23.0	24.5	18.3
**Presence of mental and physical impairment after rehabilitation (%)**					
2006	6.8	7.5	11.9	11.9	6.4
2007	7.1	8.6	13.4	13.3	7.1
2008	7.1	8.2	13.6	12.8	6.0
2010	7.7	9.4	12.2	14.9	7.2
2011	8.0	10.0	11.9	15.4	9.1
2012	8.0	9.5	10.1	17.5	7.5
2013	8.0	8.3	13.1	14.7	8.2
2014	8.0	10.1	9.4	16.0	7.4
**Limitations in occupational performance in terms of working hours per day (less than part-time) after rehabilitation (%)**					
2006	16.2	19.7	26.9	26.1	19.6
2007	16.2	19.5	27.2	22.4	17.8
2008	16.0	19.5	24.3	25.6	19.0
2010	16.9	22.3	27.1	26.0	18.3
2011	16.5	19.6	24.0	23.0	18.4
2012	16.4	16.9	25.5	24.3	17.7
2013	16.6	19.6	24.3	25.3	17.8
2014	16.5	21.2	25.2	23.6	18.3
**Limitations in occupational performance in terms of work capacity (only capable of performing light work) after rehabilitation (%)**					
2006	19.4	21.7	25.5	21.2	18.1
2007	18.3	18.9	24.8	18.8	15.3
2008	17.6	16.8	20.0	21.6	16.9
2010	17.3	17.7	20.7	19.6	17.6
2011	16.3	15.5	19.9	16.5	16.4
2012	15.8	15.6	21.7	16.0	15.8
2013	15.5	17.6	18.8	17.8	15.6
2014	15.3	16.4	17.4	17.3	15.4

**Fig 1:**
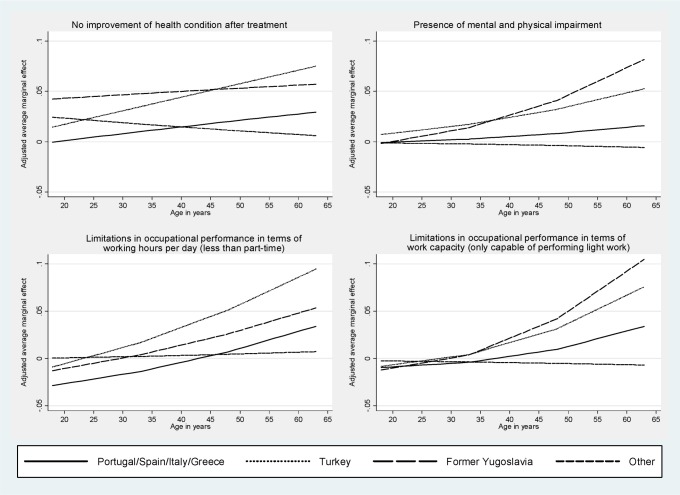
Differences in the probability for the occurrence of the four treatment outcomes as compared to German nationals by population group and age (adjusted average marginal effects estimated by means of multivariable logistic regression models with the respective outcome as the dependent variable and age-by-population group interaction terms; 10% random sample of all individuals who completed medical rehabilitation in the years 2006–2008 and 2010–2014 granted by the German Statutory Pension Insurance Scheme; cases with available data on the four outcomes studied, n = 617,683).

**Fig 2:**
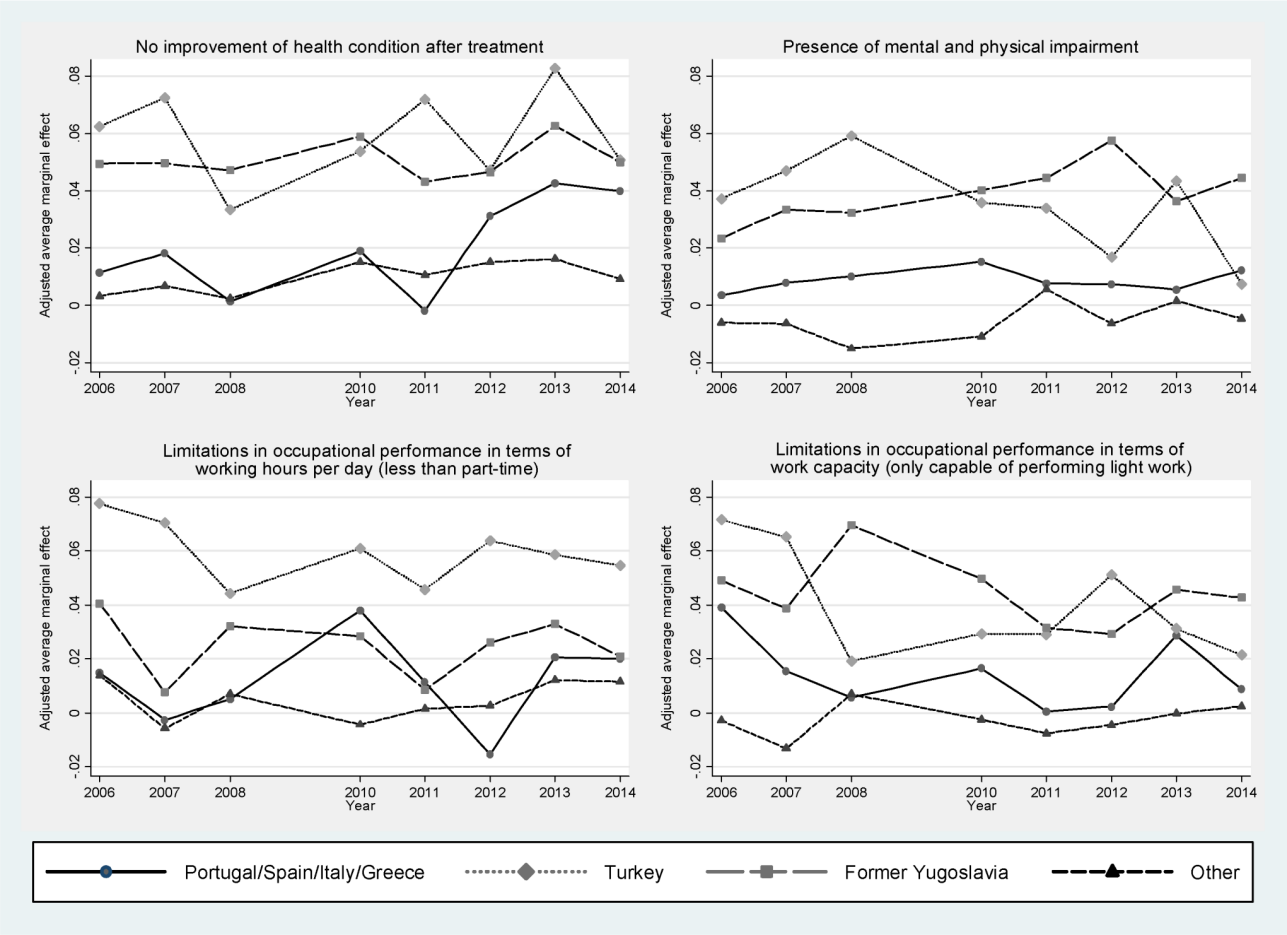
Differences in the probability for the occurrence of the four treatment outcomes as compared to German nationals by population group and year in which services were utilized (adjusted average marginal effects estimated by means of multivariable logistic regression models with the respective outcome as the dependent variable and year-by-population group interaction terms; 10% random sample of all individuals who completed medical rehabilitation in the years 2006–2008 and 2010–2014 granted by the German Statutory Pension Insurance Scheme; cases with available data on the four outcomes studied, n = 617,683; p for trend >0.05).

Also after controlling for demographic and socioeconomic factors, disparities between German nationals and the different groups of non-German nationals remained significant (Tab. 3). Nationals from Portugal/Spain/Italy/Greece, from a Former Yugoslavian country and from Turkey had a 17%, 50% and 43%, respectively, higher adjusted chance than Germans of completing rehabilitation without an improvement in their health status (adjusted odds ratio[aOR] = 1.17 [99%-CI = 1.08,1.27], aOR = 1.50 [95%-CI = 1.39,1.62] and aOR = 1.43 [99%-CI = 1.34,1.52], respectively) (Tab. 3). Non-German nationals were also more likely than Germans to have limitations with respect to their mental and physical condition despite the treatment (aOR = 1.14 [95%-CI = 1.02,1.29], aOR = 1.60 [95%-CI = 1.44,1.78] and aOR = 1.69 [99%-CI = 1.55,1.83], respectively) (Table 3). Similarly, differences in terms of occupation performance remained significant after controlling for demographic and socioeconomic confounders. This was reflected in a 11%, 61% and 23% higher adjusted chance, respectively, for being limited in terms of working hours per day (aOR = 1.11 [99%-CI = 1.01,1.21] for nationals from Portugal/Spain/Italy/Greece, aOR = 1.61 [99%-CI = 1.49,1.75] for nationals from a former Yugoslavian country and aOR = 1.23 [99%-CI = 1.15,1.32] for nationals from Turkey) and in a 8%, 35% and 37%, respectively, higher adjusted chance of being only able to perform light work (aOR = 1.11 [99%-CI = 1.02,1.22] for nationals from Portugal/Spain/Italy/Greece, aOR = 1.35 [99%-CI = 1.24,1.46] for nationals from a former Yugoslavian country and aOR = 1.37 [99%-CI = 1.28,1.47] for nationals from Turkey). Differences in the treatment outcomes between Germans and ‘other’ nationals were small and with one exception statistically non-significant. Aside from non-German nationality, a poor treatment outcome was associated with higher age, not being married, semi-skilled/unskilled labor, working part-time or being unemployed, working in manufacturing, and with a longer time absent from work in the last 12 months before treatment. Chances for a poor treatment outcome also varied with the type of diagnosis on admission to rehabilitation. While no moderation by sex could be observed, difference in the four outcomes between the population groups increased with age (Fig 1). The moderation by age was particularly pronounced for Turkish and Former Yugoslavian nationals. Disparities in treatment effectiveness did not significantly differ between the years in which services were utilized as indicated by non-significant year-by-nationality interaction terms. Consequently, average marginal effects varied only slightly and did not significantly change over the years (p for trend >0.05) (Fig 2).

**Table 3:** Results of the multivariable logistic regression models with no improvement in health condition, presence of mental and physical impairment, limitations in occupational performance in terms of working hours per day (less then part-time) and limitations in occupational performance in terms of work capacity (only capable of performing light work) after rehabilitation as the dependent variable. Adjusted odds ratios (aOR) and 99% confidence intervals [99%-CI] (10% random sample of all individuals who completed medical rehabilitation in the years 2006–2008 and 2010–2014 granted by the German Statutory Pension Insurance Scheme; cases with available data on the four outcomes studied, n = 617,683). No interaction effects included.

	No improvement in health condition		Presence of mental and physical impairment		Limitations in occupational performance in terms of working hours per day (less than part-time)		Limitations in occupational performance in terms of work capacity (only capable of performing light work)	
	aOR^1^	99%-CI	aOR	99%-CI	aOR	99%-CI	aOR	99%-CI
**Nationality (Ref.: Germany)**	1.00	1.00,1.00	1.00	1.00,1.00	1.00	1.00,1.00	1.00	1.00,1.00
Portugal/Spain/Italy/Greece	1.17	1.08,1.27	1.14	1.02,1.29	1.11	1.01,1.21	1.11	1.02,1.22
Former Yugoslavia	1.50	1.39,1.62	1.60	1.44,1.78	1.61	1.49,1.75	1.35	1.24,1.46
Turkey	1.43	1.34,1.52	1.69	1.55,1.83	1.23	1.15,1.32	1.37	1.28,1.47
Other	1.09	1.03,1.16	0.94	0.86,1.03	1.04	0.98,1.11	0.96	0.90,1.02
**Sex (Ref.: male)**	1.00	1.00,1.00	1.00	1.00,1.00	1.00	1.00,1.00	1.00	1.00,1.00
Female	1.00	0.98,1.02	1.17	1.13,1.20	0.64	0.63,0.66	1.45	1.42,1.48
**Age (in years)**	1.00	0.99,1.00	1.02	1.02,1.02	1.01	1.01,1.01	1.04	1.03,1.04
**Marital status (Ref: not married)**	1.00	1.00,1.00	1.00	1.00,1.00	1.00	1.00,1.00	1.00	1.00,1.00
Married	0.97	0.95,0.99	0.90	0.87,0.93	0.90	0.88,0.92	0.92	0.91,0.94
Other	0.95	0.88,1.01	0.73	0.67,0.80	0.58	0.54,0.63	0.68	0.64,0.73
**Occupational position (Ref: skilled labor)**	1.00	1.00,1.00	1.00	1.00,1.00	1.00	1.00,1.00	1.00	1.00,1.00
Semi-skilled/unskilled labor	1.18	1.15,1.21	1.30	1.26,1.35	1.31	1.28,1.35	1.08	1.05,1.11
Trainee/unemployed	1.07	1.01,1.12	0.96	0.90,1.03	1.22	1.15,1.28	1.07	1.02,1.13
**Employment status (Ref.: full time)**	1.00	1.00,1.00	1.00	1.00,1.00	1.00	1.00,1.00	1.00	1.00,1.00
Part-time	1.09	1.05,1.12	1.16	1.12,1.21	1.17	1.13,1.21	1.17	1.13,1.20
Unemployed	1.32	1.28,1.36	1.59	1.52,1.66	2.92	2.84,3.01	1.80	1.75,1.86
Other	1.09	1.04,1.14	1.53	1.44,1.62	1.62	1.54,1.70	1.60	1.53,1.67
**Occupation (Ref.: manufacturing)**	1.00	1.00,1.00	1.00	1.00,1.00	1.00	1.00,1.00	1.00	1.00,1.00
Services	0.99	0.97,1.02	1.03	0.99,1.07	0.85	0.83,0.88	1.13	1.10,1.16
Technical/professional	0.94	0.91,0.98	0.92	0.88,0.97	0.59	0.56,0.61	1.07	1.03,1.11
Administrative	0.96	0.93,0.99	0.95	0.91,0.99	0.45	0.44,0.47	1.40	1.35,1.44
Other	0.97	0.94,1.00	1.18	1.13,1.23	0.70	0.68,0.72	1.37	1.32,1.41
**Diagnosis (Ref: skeletal system)**	1.00	1.00,1.00	1.00	1.00,1.00	1.00	1.00,1.00	1.00	1.00,1.00
Circulatory system	0.92	0.89,0.96	4.74	4.50,4.98	1.47	1.42,1.52	1.56	1.51,1.61
Neoplasms	1.07	1.03,1.10	3.84	3.64,4.04	1.10	1.07,1.14	2.34	2.27,2.41
Mental diseases	0.90	0.87,0.92	8.49	8.15,8.84	0.93	0.90,0.96	0.51	0.49,0.52
Other	1.01	0.99,1.04	5.99	5.75,6.25	0.98	0.95,1.01	1.53	1.49,1.57
**Time absent from work in the last 12 months (%) (Ref.: None)**	1.00	1.00,1.00	1.00	1.00,1.00	1.00	1.00,1.00	1.00	1.00,1.00
<3 months	0.79	0.77,0.81	1.00	0.96,1.04	0.93	0.90,0.97	0.88	0.85,0.90
3–6 months	1.22	1.18,1.27	1.51	1.44,1.59	2.57	2.47,2.67	1.43	1.38,1.48
6+ months	1.96	1.90,2.02	2.51	2.40,2.63	5.94	5.73,6.15	2.62	2.54,2.70
Not employed	1.31	1.23,1.39	1.88	1.74,2.03	4.42	4.16,4.69	2.22	2.10,2.34

Note

^1^Odds ratio adjusted for variables in the table and federal state of residence. Ref: reference category.

## Discussion

In many countries, including Germany, immigrants encounter barriers in the health care system that may affect the outcomes of the care they receive. In order to better cater to the needs of immigrants, many health care institutions have increased their efforts in recent years to provide services which are more sensitive to the needs of an increasingly diverse population. Little is known about whether these efforts are successful and whether they contribute to reducing existing disparities in health care. The aim of this study was to examine differences in outcomes of tertiary prevention between immigrants and the autochthonous population in Germany over the period of 2006–2014. The study shows that immigrants experience poorer treatment outcomes than the majority population, with older immigrants being particularly vulnerable for a reduced effectiveness of treatment. Differences in all four outcomes were especially pronounced for nationals from Turkey and Former Yugoslavia. The observed disparities can only be partially explained by a different distribution of demographic and socioeconomic factors. This is in line with previous research that had focused on other indicators of treatment effectiveness. For example, nationals from Portugal/Spain/Italy/Greece, from Turkey and from Former Yugoslavia were reported to have a 24%, 62% and 68% higher chance of a poor self-perceived treatment outcome than Germans, independent of differences in health status as well as their demographic and socioeconomic profile (aOR = 1.24 [95%-CI = 1.12;1.37], aOR = 1.62 [95%-CI = 1.45;1.80] and aOR = 1.68 [95%-CI = 1.52;1.85], respectively) [15]. Similarly, nationals from Turkey and Former Yugoslavia are at a 70% and 41% higher risk of disability retirement despite undergoing rehabilitative treatment (adjusted hazard ratio [aHR] = 1,41 [95%-CI = 1,22; 1,64] and aHR = 1,70 [95%-CI = 1,49;1,95], respectively) [32]. These findings together with the present study suggest that immigrants experience different challenges and obstacles in the health care system that may interfere with an adequate provision of care. They arise from communication problems between patients and health care providers emerging from limited information about rehabilitative services, a reduced health literacy and poor German language proficiency [33]. These barriers make it difficult for health professionals to instruct patients about therapies and necessary exercises or to obtain their medical history, thus affecting the effectiveness of rehabilitative treatments. Aside from these challenges, problems in communication and interaction can also result from cultural needs and health care expectations that are not sufficiently address by health care providers. These, for example, comprise culture-specific illness perceptions or cultural taboos [34;35].

The present study further shows that differences between the population groups in terms of health service outcomes did not decrease over the years in which services were utilized. This suggests that measures currently implemented by health care institutions had little impact on reducing existing health care disparities between immigrants and the majority population. One potential reason is that existing approaches are unsystematic and often not properly evaluated [24]. More targeted and systematic approaches and a thorough evaluation, therefore, are needed in order to improve health care for immigrants sustainably [36;37]. One such approach is diversity management which not only allows to take into account the needs of immigrants but which also addresses disparities associated with other diversity characteristics such as sex, age and socioeconomic status [38]. Implementing diversity management in health care institutions can therefore contribute to improving health care for the entire population independently of immigrant status [39;40].

A strength of this study is the use of representative and robust routine data which allows to adjust for several confounders and to examine trends over a nine year period. However, several limitations need to be considered as well. Currently, nationality is the only available proxy to identify immigrants in routine data from the German Statutory Pension Insurance Scheme and other social security organizations in Germany. By using citizenship to define immigrants in the present study, therefore, immigrants who have a German citizenship (e.g., foreign nationals who got naturalised after migrating to Germany or those who received German citizenship at birth as children born to non-German parents residing in Germany) and who account for more than half of the population with an immigrant background, cannot be identified. This, however, can be regarded a minor limitation. Studies which were able to identify German nationals with an immigrant background in routine data by means of name-based algorithms or which used other data sources suggest that findings on non-German nationals can be extended to all immigrants residing in Germany, including those with a German citizenship. These studies also show that both groups of immigrants experience similar barriers in the rehabilitative care system [41;42]. Future research should examine whether both population groups are also similar in terms of the persistence of disparities in rehabilitative care outcomes over time.

Aside from the restriction on nationality, also the heterogeneity of immigrants in terms of levels of acculturation and German language proficiency could not be considered. Furthermore, only information on the type of diagnosis upon referral to rehabilitation and the time absent from work due to illness in the last 12 months before rehabilitation were available as proxy variables for health status. It therefore cannot be ruled out that residual confounding due to differences in health status before rehabilitation affected disparities in health outcomes between population groups. Other studies, though, which were able to take into account subjective health and the physical and mental performance before rehabilitation also identified considerable disparities in rehabilitative health care outcomes between immigrants and the autochthonous population [15]. The bias due to residual confounding by health status before rehabilitation is therefore considered to be small in the present study.

## Conclusion

The present study has implications for practice, research and policy making. It shows that current approaches and strategies of the health system to provide health care for immigrants are not sufficiently able to reduce existing disparities in health care between immigrants and the autochthonous population. Efforts therefore need to be strengthened. Systematic approaches such as diversity management can guide future activities. The findings also emphasize the need for the development of migrant-sensitive health reporting to ensure that appropriate data is collected and available in order to identify and monitor disparities in different sectors of the health system. These disparities need to be address by means of appropriate measures which need to be evaluated in terms of their effectiveness. For this purpose, also the transfer of research findings into clinical practice must be enhanced.
